# Bridging the Reciprocal Gap between Sleep and Fruit and Vegetable Consumption: A Review of the Evidence, Potential Mechanisms, Implications, and Directions for Future Work

**DOI:** 10.3390/nu11061382

**Published:** 2019-06-19

**Authors:** Essra Noorwali, Laura Hardie, Janet Cade

**Affiliations:** 1 Nutrition Epidemiology Group, School of Food Science and Nutrition, University of Leeds, Leeds LS2 9JT, UK; 2 Department of Clinical Nutrition, Faculty of Applied Medical Sciences, Umm Al-Qura University, 21421 Makkah, Saudi Arabia; 3 Division of Clinical and Population Sciences, Leeds Institute of Cardiovascular and Metabolic Medicine, School of Medicine, University of Leeds, Leeds LS2 9JT, UK; l.j.hardie@leeds.ac.uk

**Keywords:** sleep, fruits and vegetables, polyphenols, dietary intake, nutritional epidemiology

## Abstract

A substantial burden of disease and mortality globally is attributable to both sleep disruption and low intakes of fruit and vegetable (FV) and there is increasing mechanistic and epidemiological evidence to support a reciprocal relationship between the two. This review provides an overview of experimental and observational studies assessing the relations between sleep and FV consumption from 52 human adult studies. Experimental studies are currently limited and show inconsistent results. Observational studies support a non-linear association with adults sleeping the recommended 7–9 hours/day having the highest intakes of FV. The potential mechanisms linking sleep and FV consumption are highlighted. Disrupted sleep influences FV consumption through homeostatic and non-homeostatic mechanisms. Conversely, FV consumption may influence sleep through polyphenol content via several potential pathways. Few human experimental studies have examined the effects of FV items and their polyphenols on sleep and there is a need for more studies to address this. An appreciation of the relationship between sleep and FV consumption may help optimize sleep and FV consumption and may reduce the burden of chronic diseases. This review provides implications for public health and directions for future work.

## 1. Introduction

Sleep is a universal need and humans spend about one-third of their lives asleep but its function remains to be fully elucidated. Sleep health encompasses sleep architecture [1], sleep duration, quality (efficiency which is the time in bed spent asleep, sleep onset latency (SOL) which is the amount of time it takes to fall asleep) [2], timing (sleep onset is the time sleeping starts and sleep offset is waking time), variability, daytime sleepiness, and napping [3]. However, most studies have focused on sleep duration since it is easier to report accurately by participants [4]. Sleep is regulated by a two-process model that interplay akin to an hourglass timer [5]. The two-processes include process S—which is the homeostatic drive to sleep which accumulates across the day, peaks before bedtime and dissipates throughout the night—and process C which is regulated by the circadian system [6].

Sleep disruption is defined as changes in sleep continuity, timing, or duration. It is intertwined with circadian rhythm disruption and their causes could be environmental, such as shift work and jetlag, and behavioral, such as the disruption of the fasting/feeding cycle and the rest/activity cycle [7]. The National Sleep Foundation (a US non-profit organization) recommends different sleep durations for individuals according to age. Adults aged between 18–64 years are recommended to sleep 7–9 h/day [8].

### 1.1. Economic Cost of Sleep Disruption and Low Intakes of FV

Hafner and colleagues reported the economic cost of insufficient sleep from 62,000 people in the UK, US, Canada, Germany, and Japan. Insufficient sleep costs $411 billion annually for the US, $138 billion for Japan, £40 billion for UK, $60 billion for Germany, and $21 billion for Canada [9]. Sleep disruption has detrimental consequences and identifying the factors that influence it is a public health priority.

Few studies have assessed the economic cost of “unhealthy diets” that include low consumption of FV, probably due to the conceptual challenges of its definition [10]. Popkin et al. defined an “unhealthy diet” as high in saturated and trans-fat, heavy alcohol drinking, and low consumption of whole grains and FV. Using this definition, the estimated annual cost of “unhealthy diets” for China was calculated as €3.5 billion per capita [11]. The economic burden attributable to low FV consumption in Australia was estimated to be $AUS 269 million [12]. For Canada, the economic burden of inadequate consumption of FV was $CAN 3.3 billion per year, of which 30% is direct for health-care costs and 69% is indirect costs due to productivity losses [13]. The estimates of the economic cost to the NHS in the UK in 2007 was £5.8 billion for “poor diet”, the consumption of <600 g/day of FV was one aspect of “poor diet” [14].

### 1.2. Sleep Disruption and Low Intakes of FV Are Associated with Morbidity and Mortality

There is growing evidence that sleep disruption has deleterious associations for health. The Centers for Disease Control and Prevention has declared “insufficient sleep” as a public health problem because it is associated with type 2 diabetes, heart disease, obesity, and depression [15]. Short sleep duration was associated with 38% increased risk of obesity in adults from 153 prospective studies in a meta-analysis [16]. Recent evidence from other meta-analyses found that long sleep duration was associated with an increased risk of obesity [4,17]. Sleep disruption was shown to increase the risk of other diseases including; cancer [18,19,20], type 2 diabetes mellitus [4,16,21], stroke [22], cardiovascular disease, and coronary heart disease [23,24]. A consistent U-shaped association was shown between sleep duration and mortality, short and long sleep durations were associated with an increased risk of mortality [4,16,25,26,27,28]. Collectively, sleep disruption is associated with an increased risk of diseases and mortality. These associations are partly mediated through changes in dietary intake including the low consumption of FV [29], thus exploring the associations between sleep and dietary intake is fundamental.

The reciprocal relationship between sleep and diet in humans has been studied since the 1980s [30,31,32]. Sleep disruption affects dietary intake [29,33,34] and dietary intake affects sleep [2,35,36]. With the reciprocal relationship in mind, The World Health Organization (WHO) recommends consuming 400 g or more of FV per day to improve overall health and reduce the risk of chronic diseases [37]. The recommended amount of FV consumption is different between countries [38,39,40,41,42]. Despite these recommendations, FV consumption remains below the recommended levels and below the WHO recommendations in many countries [37,43,44].

Increased consumption of FV has been shown to protect against type 2 diabetes [45], coronary heart disease [46], stroke [47], and some cancers [48]. Increasing FV consumption to 600 g/day could reduce the total worldwide burden of disease by 1.8%, reduce the burden of ischemic heart disease by 31%, ischemic stroke by 19%, stomach cancer by 19%, esophageal cancer by 20%, lung cancer by 12%, and colorectal cancer by 2% [49]. Recent evidence from a dose–response meta-analysis of prospective studies reported that the consumption of 800 g/day (10 portions per day) of FV are associated with lower risks of cardiovascular diseases, cancer, and all-cause mortality [50].

A substantial burden of premature deaths globally is attributable to low consumption of FV. In 2005, total worldwide mortality attributable to inadequate consumption of FV is estimated to be up to 2.635 million deaths per year [49]. In 2013, an estimated 5.6 million premature deaths worldwide may be attributable to FV intakes below 500 g/day and 7.8 million premature deaths to FV intakes below 800 g/day [50]. In 2017, an estimated 3.9 million deaths worldwide were attributable to inadequate FV consumption according to WHO [51]. These studies highlight the importance of FV consumption thus, identifying lifestyle factors which may influence FV intakes is a public health priority.

It is clear that both sleep disruption and low consumption of FV are economically burdensome and are attributable factors to morbidity and mortality. Consequently, bridging the scientific gap between them is essential and may have key public health implications. The aim of this review is to summarize the results from experimental and observational adult human studies assessing the relationship between sleep and FV consumption. Results from animal and in vitro studies are also included to support the potential mechanisms involved. This review will also highlight implications for public health and directions for future work. We used Medline, EMBASE, CINAHL, Cochrane, and PubMed databases (see Supplementary Material Table S1 for search terms used) to find published studies exploring the relationships between sleep and FV consumption. Hand searches of reference lists of retrieved articles were also undertaken. A total of 52 human studies were found and discussed below.

## 2. Sleep and Fruit and Vegetable Consumption

Several child and adolescent studies have assessed the association between sleep measures and dietary intake including FV consumption [52,53,54,55,56,57,58,59,60,61,62,63,64,65]. The association was shown to be positive in a recent meta-analysis [66]. Short sleep duration was associated with lower consumption of FV and an increased consumption of FV in children was associated with sleeping adequately. The associations between sleep measures and FV consumption are more consistent in children, however they are not well characterized in adults [29]. Sleep requirements differ between children, adolescents, and adults [8] and there is a need for more studies to assess this relationship in adults.

Experimental and observational adult studies assessing the association between sleep measures and FV consumption are summarized in Table 1 and are explained in detail in Table 2. Fifty-two studies were identified with only 10 experimental (interventional) studies including either sleep restriction or extension [67,68,69,70] or the effects of FV items on sleep measures [71,72,73,74,75,76] (Table 1).

### 2.1. Sleep Affects FV Consumption: Experimental Studies

Sleep restriction and extension (increasing sleep duration) studies and their effects on FV consumption are summarized in Table 2. Sleep restriction in young healthy men increased appetite for FV by 17% for fruit and fruit juices and 21% for vegetables compared to sleep extension [67]. In contrast, sleep restriction had no effect on healthy snack intake composed of 1 piece of fresh fruit and 1 packet of 40 g of dried fruit and nuts in healthy Australian men [68]. Similarly, calories consumed from FV and salad did not differ between sleep restriction and baseline. However, there was an interaction between race and sleep for FV intakes and salad with African Americans consuming fewer calories from FV and salad during baseline but it did not differ from whites during sleep restriction [69]. Tasali and colleagues studied the effects of sleep extension using a home based approach in 10 overweight adults on the desire for various foods including FV, however, the study did not have a control group. Sleep extension did not change the desire for FV [70]. There is a need for more experimental studies to clarify the effects of sleep disruption on FV consumption.

### 2.2. Fruit Affects Sleep: Experimental Studies

Few studies assessed the effects of tart cherry juice and products [71,72,73,75] and kiwifruit [74] on sleep measures. However some studies had no control group to compare the effects of cherry [71,72] and kiwifruit [74] on sleep measures, whereas other studies included a control group [73,75,76]. The previous studies included a small sample size and a short period of intervention and did not meet the scoring of methodological quality to be included in a systematic review of dietary interventions targeting sleep behavior [77]. There is a need for more interventional studies to identify the effects of FV on sleep measures.

### 2.3. Observational Studies

Table 1 shows that all studies included were cross-sectional apart from two prospective studies [78,79] that had different objectives, including assessment of the association between sleep duration and lifestyle factors [78] and sleep quality and survival in elderly [79]. Most of the studies were conducted in US populations and only two observational studies had their primary objective to assess the association between sleep duration and FV consumption in pregnant women [80] and Chinese older adults (≥65 years) [81]. We conducted the other two prospective studies between sleep duration and FV consumption in UK adults [82,83]. Testing for non-linear associations has been recommended between sleep measures and dietary intakes [29], however—apart from our studies [82,84]—no study assessed non-linear associations (Table 1). We showed that sleep duration (exposure) was non-linearly associated with FV consumption (outcome) with short and long sleepers consuming less FV compared to those sleeping 7–8 h/day in a representative sample of UK adults [84]. This study strengthens the notion that people sleeping the recommended hours have a healthier lifestyle compared to short and long sleepers [85,86,87,88,89]. Potter et al. used the same dataset and found no association between sleep duration and FV consumption [90], this may be because non-linear associations were not explored between sleep duration and FV consumption. Our study [84] reinforces the need for non-linear exploration between sleep and diet in future studies.

Causal relationships cannot be inferred from cross-sectional studies and prospective studies help to clarify associations. Among UK adults, no study has assessed the associations between sleep duration and FV consumption, as well as the non-linear associations. Therefore, we addressed this question by exploring the non-linear prospective associations between sleep duration and FV consumption using a large cohort (~13,000 women) namely the UK Women’s Cohort study (UKWCS) [82]. Interestingly, cross-sectional and prospective analyses were consistent with the National Diet and Nutrition Survey (NDNS) analyses [84]. Although sleep duration was categorized differently than the NDNS analyses due to different sample sizes, we used a continuous variable of sleep duration to assess the non-linear associations in both studies and modelled this association using restricted cubic splines. Additionally, both studies assessed FV consumption using a four-day food diary and self-report of sleep duration providing more consistency. Interestingly, our prospective analyses [82] confirmed the cross-sectional associations [84] with those sleeping the recommended hours (~7–9 h/day) having the highest intakes of FV. These findings add a novel association to the literature and provide new insights to consider in experimental studies addressing the relationship between sleep and diet.

### 2.4. Studies Supporting the Inverse U-shaped Association between Sleep Duration and FV Consumption

The inverse U-shaped association we found between sleep duration and FV consumption [82,84] may be supported by the U-shaped association found in other studies between sleep disruption and unfavourable behaviors and characteristics. In a representative sample of US adults, sleep complaints were associated with sleep duration in a U-shaped relationship. Short sleepers and long sleepers reported sleep problems and those sleeping 7–8 h reported fewer sleep problems [87]. Other characteristics including smoking, alcohol drinking, and physical inactivity were associated with short and long sleep durations [85,86]. This was also shown in Swedish women with short and long sleepers being physically inactive, smokers, physiologically distressed, and having increased waist circumference compared to normal sleepers [88]. Both short and long sleep duration were negatively associated with education level, family income, leisure-time and physical activity in Chinese women [89] and a large Chinese adult population [91]. In Japanese adults, the U-shaped association between sleep duration and health were explained by the U-shaped association between sleep duration and disrupted sleep with psychosocial stress from work and family life. Short sleep duration was associated with long work hours and high work–family conflict, whereas long sleep was associated with daily alcohol drinking. Participants sleeping ~8 h had the lowest prevalence of poor sleep and unfavorable behaviors and characteristics [92]. Interestingly, the U-shaped association was found between sleep duration and serum lipid profiles in Chinese women [93], between sleep duration and diabetic retinopathy [94], and sleep duration and the risk of falls [95].

Overall, the inverse U-shaped associations observed in the previous studies may explain our findings of the inverse U-shaped association between sleep duration and FV consumption. A nutritious diet including high intakes of FV are considered one of the main keys to a healthy lifestyle [96]. Therefore, the previous studies showed that sleeping the recommended hours is associated with a healthier lifestyle, supporting our findings of higher intakes of FV in participants sleeping ~7–9 h/day. The association between sleep disruption and FV consumption may be part of the complex puzzle of the U-shaped association between sleep measures, morbidity, and mortality. Future research exploring whether FV consumption acts as a mediator between sleep disruption and morbidity is necessary to clarify the underlying mechanisms.

## 3. Chronotype and Fruit and Vegetable Consumption

Chronotype has been defined as “An individual’s phase angle of entrainment (for example, the timing of core body temperature nadir relative to dawn)” [7] which is the preference in timing of activity and sleep referred to as morning or evening type [131]. Chronotype has been assessed by various methods such as the Horne and Östberg’s Morningness–Eveningness Questionnaire (MEQ) [132]. However, the main limitation of MEQ is the unavailability of sleep timing estimates. This has been developed to the Munich Chronotype Questionnaire (MCTQ) [131] that uses mid-sleep time on non-work days as an estimate of chronotype after correcting for sleep debt on work days.

Chronotype determinants include genetic (non-modifiable) and environmental factors (modifiable) [133]. Non-modifiable determinants include rare cases of chronotype disorders such as advanced sleep-phase syndrome [134,135]. Other non-modifiable determinants include race [136], sex [137], and age [138]. Environmental factors that influence chronotype include light exposure, social interactions, urban/rural areas, and variations in the LD cycle across different latitudes and time zones [133].

Later chronotype (evening type) has been associated with less healthy behaviors such as smoking [109], physical inactivity with sedentary behavior [117], and consuming more alcohol and caffeine (from coffee and cola) compared to early chronotypes [139]. Later chronotype was associated with higher risks of some diseases such as CVD [140] type 2 diabetes [141], metabolic disorders [142], bipolar disorder [143], and obesity [117]. In a recent study conducted using the UK Biobank, a large prospective population based cohort study including 433,268 adults, later chronotype was associated with higher odds of psychological disorders, diabetes, neurological disorders, gastrointestinal disorders, and respiratory disorders. Additionally, later chronotype was associated with an increased risk of all-cause mortality compared to earlier chronotype [144]. These findings are of concern to public health and thus studies assessing the associations between chronotype and other lifestyle behaviors such as FV consumption are necessary.

Inadequate intakes of FV were associated with later chronotype in a cross-sectional study in UK adolescents [59] and US adolescents [145]. In other cross-sectional studies, later chronotype assessed by MEQ and MCTQ was associated with lower intakes of vegetables in Japanese women [146,147]. Similarly, later chronotype was associated with lower intakes of green, yellow, white vegetables, and fruits in Japanese nurses [148]. A representative sample of Finnish adults showed that later chronotypes assessed by a shortened version of MEQ consumed less fruit [149].

Patterson et al. found that early chronotypes consumed more servings of FV compared to later chronotypes in UK adults from the UK Biobank project [109]. Chronotype was self-reported by asking participants “Do you consider yourself to be (1) definitely a morning person, (2) more a morning than an evening person, (3) more an evening than a morning person, (4) definitely an evening person”. This was consistent with another recent study conducted by Patterson et al. using the UK Biobank data with a difference of including sleep duration and chronotype as independent variables suggesting an interactive effects between sleep homeostatic and circadian influence. Later chronotype and longer sleep was associated with higher odds of consuming <5 servings/days of FV compared with adequate sleep and earlier chronotype. However, earlier chronotypes and adequate sleep was associated with lower odds for all cardiovascular risk behaviors including tobacco use, physical inactivity, highly sedentary behavior, and overweight/obesity except FV consumption <5 servings/day [117].

In contrast, no association was found between chronotype and vegetables and salad in German adolescents [150]. Earlier chronotype assessed by MEQ was associated with lower intakes of vegetables and no association with fruit intake [151]. No association was found between chronotype and FV consumption among Brazilian undergraduate students [110].

The previous studies show that later chronotypes tend to consume unhealthy diets with low intakes of FV. However, the results are contradictory and a main limitation of the previous studies is the lack of usage of objective methods to measure chronotype such as actimetry and validated dietary assessment methods. There is a necessity to assess the associations between chronotype and FV consumption using validated objective methods.

## 4. Mechanisms for the Relationship between Sleep and Fruit and Vegetable Consumption

**Figure 1:**
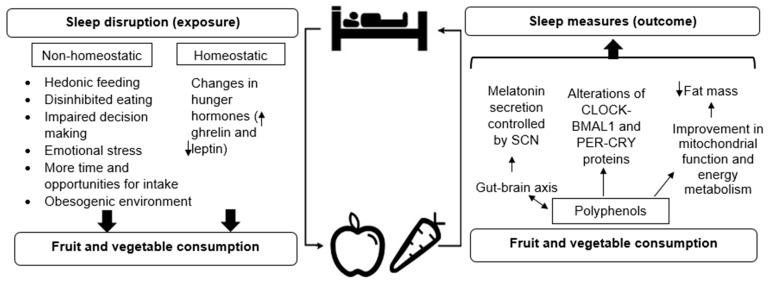
Potential reciprocal mechanisms between sleep duration and fruit and vegetable consumption. Sleep disruption may influence dietary intake through non-homeostatic and homeostatic mechanisms. On the other hand, FV consumption may influence sleep through their polyphenol content through several potential pathways. With further research, other potential mechanisms may be identified. Legend: SCN (suprachiasmatic nuclei); CLOCK (circadian locomotor output cycles kaput); BMAL1 (brain and muscle aryl hydrocarbon receptor nuclear translocator-like 1); PER (period); CRY (cryptochrome).

The potential mechanisms underlying the reciprocal relationship between sleep and FV consumption are shown in Figure 1. Several mechanisms have been proposed of the reciprocal relationship between sleep disruption and dietary intake that may subsequently lead to obesity and metabolic diseases [35,152,153,154,155,156,157].

### 4.1. Homeostatic Mechanisms

Sleep disruption may influence dietary intake through non-homeostatic and homeostatic mechanisms (Figure 1). Homeostatic mechanisms include energy homeostasis mediated by satiety hormonal changes ghrelin and leptin. Leptin sends satiety signals to the appetite control centers in the brain and ghrelin sends signals from the stomach to the brain stimulating an increase in appetite [158].

A number of studies have observed associations between sleep disruption on leptin and ghrelin levels. In a laboratory study on 10 healthy men, Mullington et al. observed a reduction in diurnal amplitude of leptin during the days of sleep deprivation [159]. Interestingly, amplitudes of leptin returned to normal in the period of sleep recovery. Similarly, leptin levels decreased when sleep was restricted to 4 h in 11 adults [160]. Furthermore, sleep restriction reduced leptin by 18% and increased ghrelin by 28% in 12 healthy men [67]. Other laboratory studies indicated an increase of ghrelin after sleep restriction [161,162,163,164]. However, the effects of sleep restriction on ghrelin and leptin are contradictory [164,165,166,167,168,169] with a suggestion of sex differences [170]. The variability in ghrelin and leptin responses to sleep restriction may be due to the small sample sizes, differences in timing of blood chemistry and analyses and variability in sleep restriction hours.

With respect to sleep and FV consumption mechanisms, laboratory studies showed that disrupted sleep changes appetite-related hormones ghrelin and leptin, which may increase the preference for energy-dense foods [33] probably leading to lower consumption of FV.

### 4.2. Non-Homeostatic Mechanisms

Non-homeostatic mechanisms have been supported with observational and experimental studies [171]. In a meta-analysis, sleep deprivation was one of the most prominent lifestyle determinants of increased food intake [172]. People eat more after sleep loss to compensate for the additional energetic cost of wakefulness [173]. Consistently, sleep deprivation increased food purchasing in men with preference to energy-dense, rewarding foods [174]. This preference for energy-dense foods may potentially lead to lower intakes of FV. Recent evidence suggests that similar to sleep restriction, long sleep duration may impair energy homeostasis through unhealthy dietary choices, leading to potentially lower intakes of FV [175].

Non-homeostatic mechanisms linking sleep disruption with FV consumption include hedonic feeding (Figure 1), which is the consumption of food to obtain pleasure in the absence of energy deficit [176]. To study the effects of sleep disruption on non-homeostatic reward-driven behavior, brain imaging studies were conducted supporting the non-homeostatic hypothesis [177]. After one night of sleep deprivation, brain activity changed in response to food stimuli and was associated with an increase in appetite [178]. Furthermore, sleep restriction to 4 h for 6 days increased the neuronal response to food stimuli and activated brain regions associated with reward [179].

Daytime sleepiness reduced the activation of ventromedial prefrontal cortex, a brain region involved in the ability to inhibit and control emotions and behavior, when participants were shown “high calorie food” compared to “low calorie food” images (included fresh salad and FV). Additionally, this reduction in prefrontal activation predicted over-eating in women [180]. Sleep restriction increased the neuronal response to “unhealthy” food images compared with “healthy” food images (that included FV) [181]. Consistently, following sleep restriction, appetite sensations and food reward increased compared to controls [182]. The previous brain imaging and experimental studies of sleep restriction provide some non-homeostatic mechanisms for sleep disruption, enhancing hedonic stimulus processing in the brain and altering brain connectivity leading to food reward, food craving, and affecting food decisions. The enhanced reward mechanism may promote energy-dense food consumption, leading to lower intakes of FV.

It has been shown that high disinhibited eating (tendency toward overeating in response to different stimuli; for example the presence of palatable food or emotional stress [183]) mediated the relationship between disrupted sleep and weight gain [184,185,186]. The mediating effect of disinhibition between disrupted sleep (short/long sleep durations, poor sleep quality) and weight status may be due to over-eating and less healthful food choices [187]. In a cross-sectional study of 187 women and their children, disinhibition scores (higher scores indicate higher disinhibition) _were negatively associated with FV consumption in both mothers and their children [188]. Consistently, in a prospective study of 2 year follow-up of men, disinhibition scores were negatively associated with fruit intake [189]. These studies provide evidence that sleep disruption may lower the intakes of FV through the mediating effects of disinhibited eating.

Furthermore, emotional eating and stress were shown to influence the association between sleep duration and dietary intake [190]. Disrupted sleep increases emotional reactivity [191] leading to an increase in dietary intake specifically energy-dense foods to improve the mood and stress of individuals with their pleasing effects through the opioidergic, dopaminergic, and serotonergic systems [192] resulting in potentially lower intakes of FV. Sleep disruption deficits impulse control [193] that plays a major role in inhibiting appetitive thoughts and behaviors [156], when impulse control is altered this results in impaired decision making leading to excess dietary intake, energy-dense foods for reward and potentially lower intakes of FV. Sleep disruption accompanied with an obesogenic environment—“ the sum of influences that the surroundings, opportunities, or conditions of life have on promoting obesity in individuals or populations” [194]—may enhance behaviors including irregular eating with fewer main meals, more frequent energy-dense snacking, and altered time of intake leading to potentially lower intakes of FV [29].

### 4.3. Mechanisms for Effects of Polyphenols on Sleep

#### 4.3.1. Animal Studies

With the reciprocal relationship between sleep and dietary intake in mind, FV consumption may influence sleep measures through their polyphenol content through several potential pathways. Polyphenols are phytochemicals that are abundant in our diets and have a probable preventive role from CVD [195], ischemic heart disease [196], stroke [197], and cancer [198]. Polyphenol profiles are complex in foods and mostly contain multiple classes of polyphenols in a single plant. The main sources of polyphenols are FV, tea, coffee, red wine, cereals, grains, and soy beans however, bioavailability differ extremely between the various polyphenols. Polyphenols are classified and sub-classified based on the number of phenol rings that they contain and of the structural elements that bind these rings to one another. The main classes of polyphenols are flavonoids, phenolic acids, stilbenes, lignans, and other polyphenols [199].

The direct and indirect effects of flavonoids in the brain including cerebrovascular blood flow and synaptic plasticity that improve learning and memory have been previously reviewed [200] and the role of sleep on memory has been highlighted [201]; however, there is a lack of studies linking sleep with polyphenols. Some animal studies (Table 3) have investigated the effects of different types of polyphenols on clock genes, circadian rhythms, and sleep/wake cycle with few studies conducted in humans (see Section 4.3.2).

The first potential mechanism of how polyphenols from FV consumption may affect sleep measures is through the gut–brain axis (Figure 1) via serotonin and γ-aminobutyric acid (GABA) receptors, consequently affecting nocturnal secretion of melatonin. Spinosin, a C-glycoside flavonoid of semen Ziziphi spinosae, a herb that has been used to treat insomnia and other diseases, reduced SOL and increased non-rapid eye movement sleep and sleep duration and increased rapid-eye movement sleep time via serotonin 1A receptor (5-hydroxytryptamine, 5-HT_1A_) in Male Sprague–Dawley rats [202].

Other studies found that different polyphenols modulated sleep via GABA receptors. Polyphenols such as phlorotannins [203,204] and triphlorethol A (seaweed polyphenols) [216], red cabbage extracts [217], and kiwifruit extracts [206] decreased SOL and increased sleep duration via GABA receptors in mice. Other polyphenols such as bioflavonoids extracts from *Rhus parviflora* referred as Tintidikah, a medicinal plant used in south Asia, were the most potent components in decreasing SOL and increasing sleep duration via GABA receptors [205]. Seeds of *Ziziphus mauritiana*, a hypnotic widely used in Asian countries, contained flavonoids and phenolic acids that increased sleep duration in mice administered with sodium pentobarbital [220]. Furthermore, the seed and leaf extracts derived from romaine lettuce potentiated the pentobarbital-induced sleeping behavior in mice [218]. In contrast, GABA in black tea did not decrease SOL induced by sodium barbital—a hypnotic—in mice, but SOL was decreased and sleep duration was increased with sodium pentobarbital, a hypnotic [207]. Collectively, the previous animal studies found that different polyphenols via serotonin and GABA receptors decreased SOL and increased sleep duration however, further human studies are required to confirm this.

Since the circadian system and their clock genes are intertwined with the sleep/wake cycle [7], the second potential mechanism of how polyphenols derived from FV consumption may influence sleep is through their effects on circadian rhythms, clock gene expression, and peripheral clocks (Figure 1). An animal study investigated the effects of resveratrol, a dietary polyphenol present in a variety of foods including FV, on circadian period and body temperature [209]. Compared to controls, resveratrol supplementation for 2 weeks in constant dark condition in primate grey mouse lemur shortened free-running period, reduced mean body temperature and locomotor activity indicating that resveratrol supplementation influences the circadian clock of those animals. Limitations of the study including the short intervention period and small number of mice (*n* = 13) requires further exploration. However, Pifferi et al. extended the resveratrol supplementation for 4 weeks in another study [210] and observed a reduction of locomotor activity onset in dark conditions, suggesting a better synchronization.

The effects of resveratrol supplementation on clock genes was investigated in several animal studies. The expression of clock genes Period (PER) 1, PER 2, and brain and muscle aryl hydrocarbon receptor nuclear translocator-like 1 (BMAL1) were increased in cultured Rat-1 cells with resveratrol for 8 h [212]. Resveratrol reversed the change induced by high-fat feeding in the expression of reverse erythroblastosis (REV-Erbα), a nuclear receptor, in adipose tissue indicating that resveratrol polyphenol targets the clock genes and thus influences sleep [213].

Pifferi et al. observed an increased proportion of active-wake time during the resting phase (light) of the sleep/wake cycle after 3 weeks of resveratrol supplementation in mice. Negligible changes in active-wake time during the active phase (dark) of the sleep wake cycle suggested that resveratrol activity depends largely on the time of administration [219]. This was consistent with another study that noted that resveratrol administration on male rats behaved as an antioxidant during the night and as a pro-oxidant during day-time [223].

Furthermore, grape seed proanthocyanidin extract (GSPE) treatment maintained nocturnal melatonin levels and modulated the circadian rhythms when it was administered at the start of the day, rather than at night [211]. GSPE administration for 21 days in healthy rats and in rats with diet-induced obesity, clock genes were overexpressed positively with a dose-dependent manner. In addition, BMAL1 protein increased and PER 2 was overexpressed whereas Rev-Erbα was repressed in the liver, gut, and white adipose tissue in healthy rats. This was also observed in the liver and gut of diet-induced obesity rats [214]. GSPE administration modulated clock genes in rat liver by increasing BMAL1 only when administered when the light were turned off suggesting also time-dependency effects [215]. The effectiveness of polyphenols during periods of the day could be due to the discrepant functionality of the suprachiasmatic nuclei (SCN). It has been shown that SCN cells are extensively coupled during the day, when the cells exhibit synchronous neural activity, but minimally coupled during the night, when the cells are electrically silent [224].

Tea polyphenols were capable of manipulating circadian clock genes by enhancing BMAL1 and ameliorated neural redox imbalance and mitochondrial dysfunction [222]. The intake of cherry nutraceutical product decreased diurnal activity and increased nocturnal activity in young and old rats (representative of nocturnal animals). In contrast, the opposite effects were observed for ringdoves (representative of diurnal animals), indicating that effects are modulated depending on the nature of the animals’ circadian rhythms [208]. The previous animal studies showed that polyphenol administration modulated the circadian system through circadian rhythms, clocks, and the sleep/wake cycle with dose and time dependency and possible sex differences providing insight that polyphenols may influence sleep measures.

Since the metabolic state of a cell is coupled to the molecular clock, diet may modify rhythmic cellular activities [6]. In light of this, the third potential mechanism of how polyphenols from FV may affect sleep is by activation of pathways that promote silent mating type information regulation 2 homolog 1 (SIRT1) protein expression [225]. SIRT1 modulates the ventromedial hypothalamic clock, a brain region that contains neuronal food-synchronized clocks that contribute to regulation of the circadian rhythm in feeding behavior [226]. SIRT1 has a central role for reactive oxygen species mainly produced as a consequence of mitochondrial functions [227]. It has been identified that several polyphenols, such as resveratrol, act as dietary activators of SIRT1 [225]. In turn, SIRT1 binds CLOCK-BMAL1 and promote the degradation of PER 2 [228] thus influencing sleep. Alternatively, it has been suggested that resveratrol through its action on SIRT1 improves mitochondrial function and energy metabolism by decreasing fat mass, leading to changes in sleep [219].

The previous animal studies showed that polyphenols modulated sleep through several potential mechanisms however, there is a need for human studies to confirm these mechanisms.

#### 4.3.2. Human Experimental and Observational Studies

The effects of FV consumption on sleep may be due to their high content of melatonin and serotonin [229]. Tart cherry juice has been shown to increase urinary melatonin concentrations in humans [73]; however, this is yet to be confirmed. Alternatively, the effects of polyphenols on sleep measures may be through their antioxidant content reducing oxidative stress and improving sleep quality [2]. St-Onge suggested that plant based diets improve mitochondrial function, energy metabolism, body composition, lower body fat and abdominal adiposity, consequently this may potentially improve sleep quality [230]. However, this was not specifically for FV consumption but diets high in plants.

The effects of different polyphenols on sleep architecture and sleep measures were conducted in few human studies (Table 4). Human experimental studies provide conflicting results with some showing an improvement in sleep measures after polyphenol administration and others not showing any effects. These mixed results may be due to the diverse intervention periods, different types of polyphenols, and doses. The longest intervention period was 90 days [231] and longer intervention studies are required. Furthermore, polyphenol effects from supplements differ from their effects from foods relatively due to their bioavailability and concentration [232]. Another probable reason for the distinctive results is the small number of participants, different study designs, and participants. More effects were shown in participants reporting sleep disturbances, pre-hypertensives, and memory impairment than healthy adults. Experimental trials on participants with sleep problems differ from healthy free-living individuals; therefore, it is necessary to consider the potential for non-representative samples taking part in experimental studies.

Few observational studies have assessed the associations between isoflavones, a polyphenol mainly found in soybeans and legumes, with sleep measures [241,242]. Cui et al. assessed the cross-sectional association between isoflavone intake and self-reported sleep duration and quality in 1076 Japanese adults [241]. High intakes of isoflavones were associated with adequate sleep duration (7–8 h) and better sleep quality. In contrast, a longitudinal study showed that the highest quartile of soy isoflavone intake was associated with lower odds of long sleep duration (≥9 h/night) and lower odds of falling asleep during daytime in women only. There was a persistent inverse association between isoflavone intake and sleep duration suggesting these effects are due to the estrogenic contents of isoflavones [242]. These inverse associations were consistent with our study exploring the prospective associations between polyphenols derived from FV and sleep duration in UK women [83]. To our knowledge, our study is the first prospective study to explore associations between FV items and their polyphenol content with sleep duration.

Whilst FV consumption may have an immediate effect on sleep, it may also have a longer term impact. Greenwood et al. assessed the stability of dietary patterns in women from the UKWCS using cluster analysis at baseline and after 5 years. Results showed that there was moderate stability in dietary patterns in the UKWCS [243] and in other studies [244,245,246,247]. Thus, exploring the longitudinal associations between FV consumption and sleep duration using the UKWCS was appropriate.

## 5. Public Health Implications

With the reciprocal relationship between sleep and FV in mind, this review has two main implications that may contribute to public health. Healthy lifestyle patterns have focused mainly on dietary intake and physical activity however, recently, awareness of sleep as a healthy behavior has been raised [248,249]. A first implication, dietary guidelines could include information on sleep and chronotype. A natural starting point is improving sleep hygiene by recommending behavioral and environmental practices to promote better sleep. These practices include optimising temperature, bedding, mattresses, and sound. Sleep hygiene education have shown effective enhancement of sleep quality and decreased daytime sleepiness in adults [250,251] and children [252,253]. Dietary guidelines and nutrition professionals could promote better sleep by eliminating or reducing caffeinated foods and beverages before bedtime, smoking cessation, massage therapy, dim or reduce bright lights during dark hours, engage in physical activity throughout the day and have consistent sleeping and waking times [254].

If future studies continue to support previous findings that later chronotype initiates lower consumption of FV and less healthy behaviors, governments should revise their guidelines accordingly. Dietary recommendations tailored to late chronotypes would ultimately be another worthwhile development. Such recommendations may include pre-planning and preparation of meals to prioritize and increase the consumption of FV. Furthermore, if future studies support that the timing of FV consumption may impact sleep measures, recommendations on the optimal time of FV consumption alongside the 5-a-day guidelines [255] will be informative. However, as few human studies have addressed this question, a greater body of evidence would be required before such recommendations could be proposed.

Promoting FV consumption is a key objective of food and nutrition policy interventions conducted by governments and non-governments. The success of campaigns and interventions conducted around the world in terms of increasing the daily consumption of FV remain modest [256]. The second implication is the incorporation of sleep screening in GP practices, hospitals, weight-loss programs, and campaigns targeting higher consumption of FV. Sleep screening questions on timing, duration, difficulty falling asleep, waking up at night, refreshed feeling upon waking and sleepiness during the day should be included [254]. If desired answers are not received, further assessment can be conducted by using the Pittsburgh Sleep Quality Inventory (PSQI) [257]. Participants with an indication of poor sleep or sleep disorders should be referred to sleep clinicians.

## 6. Directions for Future Work

Exploring the links between sleep and FV consumption are scarce and future studies are required to take into account several factors (Table 5).

It would be productive to reach agreement on the best ways to assess sleep duration and chronotype. Although the recommendations for sleep duration have been provided [8], individual differences need to be considered by exploring optimal sleep duration in a laboratory. Next, the difference between optimal sleep duration and habitual sleep duration can be obtained, this difference represents potential sleep loss which is not clearly recognized by individuals. Previous findings confirmed that evaluating optimal sleep duration may be a useful clinical marker of sleep loss and individual differences [258]. However, laboratory experiments of sleep deprivation have shown that individuals have differential neurobehavioral vulnerability to sleep loss suggesting a polygenetic phenotype [259,260]. Omics (transcriptomics, epigenomics, and metabolomics) approaches were suggested as biomarkers for identifying differential vulnerability to sleep loss [261]. Identification of such markers will provide a viable means to determine those individuals who may need more habitual sleep or who may need to prevent or mitigate sleep deprivation through lifestyle choices and effective interventions and countermeasures (e.g., caffeine, naps, etc.). Variation in sleep measures and chronotype are related to circadian clock genes and non-circadian genes [262]. Sleep and diet research may need to explore the effects of sleep disruption on FV consumption based on genetic disparities.

One of our studies focused on FV items and their polyphenol content with sleep duration [83]. It would be informative to explore the effects on other sleep measures (sleep timing and quality). Furthermore, future intervention studies comparing the effects of different FV items (individually and combined) on sleep measures will be instructive and may be beneficial in identifying the underlying mechanisms. Previous studies found that activities done during the day have an effect on sleep [263,264,265,266,267]. Related to this, future studies may compare the effects of FV consumption at specified time points on sleep measures. Nevertheless, little is currently known about associations between many FV items and sleep measures.

Adherence to a Mediterranean diet (the highest tertile) was associated with better sleep quality compared to those in the lowest tertile [268]. It could be that different FV items may have different effects on sleep measures due to their moisture, water content [269], fiber, polyphenols, and antioxidants [270]. Lower consumption of dietary fiber was associated with less slow-wave sleep (deep sleep) [271,272] however, few studies explored the effects of different polyphenols on sleep architecture (see Section 4.3.2) and more studies will be instructive. People who eat several servings of FV per day, their total polyphenol intake reaches ~1 g/d [199]. The assessment of polyphenol intake is difficult to evaluate by using similar methods to dietary assessment due to their bioavailability and bio-efficacy variances [232]. Therefore, biomarkers for polyphenol exposure would be very useful. Limited studies found that food matrix affects the bioavailability of polyphenols and thus influence their absorption [273,274]. Future studies may compare the effects of different polyphenols on sleep measures between fasting participants and participants consuming a complex meal or FV items. Consideration of the dietary fiber content of FV items in these studies are necessary because dietary fiber stimulates intestinal fermentation that may influence the production of microbial metabolites that may have consequences on the absorption of polyphenols [199]. Another factor to consider in studies exploring the effects of polyphenols from FV on sleep, is controlling for other sources of high polyphenol content from foods such as coffee, tea, red wine, soy, and chocolate.

The antioxidant properties of polyphenols have been widely studied [275]. However, it is uncertain whether increased antioxidant nutrient intake or supplementation would modify sleep. Nonetheless, studies reported reduced antioxidant capacity in serum of patients with obstructive sleep apnea (OSA) [276], reduced dietary intake of antioxidants in veterans with OSA [277]. Related to OSA, participants with the metabolic syndrome had reduced serum concentrations of antioxidants [278]. Furthermore, antioxidant nutrient intake from high consumption of FV or supplement intake were proposed as potential moderators of cognitive decline and CVD from OSA [279]. In a recent cross-sectional study conducted among 3941 Korean men, short sleepers (<6 h) with low consumption of dietary antioxidant had a higher risk of obesity than those with a high consumption of dietary antioxidants [280]. These results suggest that the increased risk of obesity associated with short sleep duration may be modified by the consumption of dietary antioxidants. In light of this, as a starting point, epidemiological studies could subgroup FV based on similarity of total antioxidant capacity to explore their relationship with sleep. Ten subgroups of FV were proposed based on food component and classification variables (botanic family, plant part, color, and total antioxidant capacity) [281]. Furthermore, long-term prospective randomized controlled clinical trials can study the effects of antioxidants from FV combined based on the 10 groups, or antioxidants from supplements in individuals with short, recommended, and long sleep duration. In addition, other sleep measures (sleep architecture, quality, and timing) could be explored in relation to antioxidant intake. Objective markers of antioxidant intake and oxidative stress should be initiated and compared across persons with short, recommended, and long sleep durations, and also compared across persons with poor/good sleep quality and those with different chronotypes. In those randomized controlled trials, it is necessary to select FV that undertaken similar food processing and handling methods to overcome their effects on preservation of antioxidants [282].

Sleep restriction studies (Table 2) provide conflicting results on their effects on FV consumption and there are a lack of studies assessing the timing of sleep in relation to FV consumption, more studies investigating the timing of sleep and taking into account chronotype are required to clarify this relationship. Studies exploring the effects of sleep extension on FV consumption (Table 1) are few, more studies are needed to help clarify the underlying mechanisms between sleep disruption and FV consumption. Non-linear relationships between FV and sleep need to be considered in sleep restriction/extension studies. A recent study showed the feasibility of extending sleep by sleep hygiene intervention and their effects on dietary intake and energy balance [283]. The results showed that sleep extension led to reduced intakes of free sugars compared to controls. It would be interesting to conduct a similar study and explore the effects of sleep extension in habitual short sleepers on FV consumption. One factor that may influence the effects of sleep extension interventions on diet is chronotype. Therefore, comparing the effects of sleep restriction/extension on FV consumption between different chronotypes is essential.

Additional studies of effects of sleep restriction/extension on FV consumption in different populations would also be useful. Differences in sleep between different races and ethnicities have been reported. Black individuals tend to have shorter sleep durations and poorer sleep quality than white individuals [284,285,286,287]. It has been proposed that the underlying mechanisms of ethnic/racial disparities in sleep include several potential biological, psycho-behavioral, sociocultural, and environmental factors [286,287]. These potential mediators are important to explore in future research conducted in different race/ethnic individuals assessing associations between sleep measures and FV consumption. Other populations to consider could include elderly people, clinical populations, shift workers, and less-developed countries. Little is known about sleep and FV consumption in these people, to my knowledge.

A study explored the effects of short and long sleep durations on taste preference in healthy adults. Habitual long-sleepers preferred sweeter stimuli following sleep restriction while sleep extension did not change taste preference in habitual short-sleepers [288]. However, in adolescents, sweet foods were more appealing after sleep restriction [289]. This may be an explanation for the low intakes of FV in short and long sleepers in our studies [82,84]. Because FV require some preparation, convenience and time are important factors today as the pace of life has increased, therefore, people tend to buy products that require minimum preparation [256]. In light of this, the question of whether short and long sleepers substitute FV with off the shelf and convenient desserts and sweets is not well understood and will be a valuable point of inquiry to address in future studies that assess the association between sleep measures and FV consumption.

Reciprocity between sleep and exercise exists; sleep disruption could impair an individual’s capacity for exercise and increase the risk of exercise-induced injuries, conversely, acute and regular exercise effect sleep architecture and measures depending on numerous factors; sex, age, fitness level, BMI, intensity and duration of exercise, time of day and environment (indoor or outdoor) [290]. The results are conflicting and effects of exercise on sleep were mostly shown in people with sleep disorders and trivial improvement in sleep in individuals with good sleep [254,290]. There is not enough evidence that exercise may effect FV consumption and there is a paucity of human studies on this subject, it is important to study how to optimize exercise protocols to increase FV consumption and improve sleep.

Studying the complex relationship between sleep and diet needs to take into consideration numerous components. Current usage of digital devices is unprecedented and keeping pace with the digital revolution we are experiencing is fundamental. Future sleep extension studies need to compare between the effects of digital cognitive behavioral therapy [291] and sleep hygiene education on sleep, quality of life, and psychological well-being. Another factor to consider in future studies is stress. Mental stress and depression have increased dramatically over the last 50 years. Stress has been found to be associated with disrupted sleep and increase desire for palatable food, thus causing obesity. On the other hand, improving sleep patterns and nutritional status may reduce the severity of stress and mental disorders [292]. This highlights the importance of the need to further examine the complex relationship between sleep, diet, and stress. Related to this, sleep and self-control are intertwined, sleep disrupted individuals are at an increased risk of impaired decision making, including dietary selections [293]. Self-control has two effects on healthy behavior (such as physical activity, eating healthily (high intakes of FV for example), reducing alcohol intake and not smoking); an indirect effect mediated by intentions and a moderated effect on the intention–health behavior relationship. Hagger et al. proposed several pathways of how sleep may affect health behavior in the context of the health self-control model [294]. People with better sleep quality and sufficient duration will be more likely to be able to form intentions to engage in healthy behaviors. The underlying mechanism for this effect is because better sleep (quality and quantity) provide individuals with sufficient cognitive resources for more effective planning. In light of this, does self-control have a role in the association between sleep duration and FV consumption? Future studies addressing this will be extremely valuable in clarifying the underlying mechanisms.

## 7. Conclusions

Based on health psychology research from five decades, a nutritious diet and sleep moderation and optimism are two of the main keys to a long, happy, healthy, and productive life [96]. The substantial attribution of both sleep disruption and low intakes of FV on the global burden of diseases are well documented and understanding the reciprocal relationship between them is necessary. In this review, we have provided epidemiological evidence (cross-sectional and prospective) in adults that sleep duration is non-linearly associated with FV consumption with short and long sleepers consuming less FV and sleeping the recommended ~7–9 h/day is associated with higher intakes of FV in a sub-group of UK adults. Experimental studies are limited and there is a need for robust intervention studies of sleep (restriction/extension) on FV consumption and also intervention studies of the effects of FV consumption on sleep.

This review provided potential reciprocal mechanisms linking sleep disruption with FV consumption. Disrupted sleep may influence FV consumption through homeostatic and non-homeostatic mechanisms. On the other hand, FV consumption and their polyphenol content may alter sleep measures through the gut–brain axis, their influence on circadian rhythms; clock gene expression and peripheral clocks, and by the improvement of mitochondrial function and energy metabolism by decreasing fat mass leading to changes in sleep, unidentified mechanisms may exist. With further research, interactions between sleep measures and FV consumption may be clarified and potentially reduce the burden of chronic diseases and premature deaths.

## Figures and Tables

**Table 1:** Summary of human studies [references] assessing the association between sleep and FV consumption in adults.

	Study Type				
	Observational		Experimental (Intervention)		
	Cross-Sectional	Prospective	Sleep Restriction	Sleep Extension	Fruit Intervention
**Exposure/outcome not clearly stated**	[89,97,98,99,100]				
**Exposure**					
Sleep	[82,84,90,101,102,103,104,105,106,107,108,109,110,111,112,113,114,115,116,117,118,119,120,121]	[78,79,82]	[67,68,69]	[67,70]	
Diet including FV	[80,81,122,123,124,125,126,127,128,129]	[83]			[71,72,73,74,75,76]
**Outcome**					
Sleep	[80,81,122,123,124,125,126,127,128,129]	[83]			[71,72,73,74,75,76]
Diet including FV	[82,84,90,101,102,103,104,105,106,107,108,109,110,111,112,113,114,115,116,117,118,119,120,121]	[78,79,82]	[67,68,69]	[67,70]	
**Populations**					
UK population	[82,84,90,109,117]	[82,83]			[75]
US population	[80,97,99,101,102,103,104,105,107,108,111,114,123,125,127]		[67,69]	[67,70]	[73]
Other populations	[81,89,98,100,106,110,112,113,115,116,118,119,120,121,122,124,126,128,129]	[78,79]	[68]		[71,72,74,76]
**Sleep assessment**					
Subjective	[80,81,82,84,89,90,97,98,99,100,101,102,103,105,106,107,108,109,111,112,113,115,116,117,118,119,120,121,122,123,124,125,126,127,128,129]	[78,79,82,83]			[73]
Objective	[104,114]		[67,68,69]	[67,70]	[71,72,74,75,76]
**Sleep measurements**					
Sleep duration	[80,81,82,84,89,90,97,99,100,101,102,105,106,107,108,109,110,111,112,113,114,115,116,117,118,120,121,123,125,126,127,128,129]	[78,82,83]	[67,68,69]	[67,70]	[71,72,73,74,75,76]
Sleep quality	[81,98,100,103,108,113,114,119,120,121,122,123,124,126]	[79]			[71,72,73,75,76]
Sleep timing	[104]				
**Associations between sleep and FV**					
Significant association	[80,81,82,84,89,97,99,100,101,102,103,104,105,106,109,111,114,115,116,117,118,120,122,123,124,126,128,129]	[78,79,82,83]	[67]		[71,72,73,74,75,76]
No association	[90,98,107,108,110,112,113,119,121,125,127]		[68,69]	[67,70]	
**No control group**				[70]	[71,72,74]
**Primary objective of study was to assess associations between sleep and FV**	[80,81,82,84]	[82,83]			[71,72,73,74,75,76]
**Assessed non-linear associations between sleep and FV**	[82,84]	[82]			

Legend: FV (fruits and vegetables), UK (United Kingdom), US (United States).

**Table 2:** Adult human studies assessing the relationship between sleep measures and fruit and vegetable consumption.

Author, Year (Ref)	Country	Population	Sample *n*	Exposure	Outcome	Adjusted Variables	Findings Reported on Sleep and FV	Comments
**Cross-sectional studies**								
Patel et al., 2006 [97]	United States	Nurse’s Health study	68,183	Subjective report of sleep duration. Sleep duration categorized to ≤5 h, 6 h, 7 h, 8 h, and ≥9 h	FFQ	No adjustment	FV consumption differed between sleep duration categories in baseline characteristics	Exposure and outcome not clearly stated. The significant difference in FV consumption between sleep duration groups could be due to the numerous categories of sleep duration
Adams and Colner 2008 [101]	United States	College students aged 18–25 years	40,209	Subjective report of sleep duration	FV consumption (servings/d)	Not clear	Sleep duration was a significant predictor for FV intakes, increased FV intake was positively associated with sleep duration	Sleep duration was combined in a physical health model based on health issues identified by the Centers for Disease Control and Prevention
Stamatakis and Brownson 2008 [102]	United States	Participants aged 20–92 from rural communities in Missouri, Tennessee, and Arkansas	1203	Subjective report of sleep duration. Sleep duration categorized to <7 h, 7–9 h, and ≥ 9 h	Self-report of FV consumption (servings/d) over the past month	Age, sex, ethnicity, education, marital status, and household income	Short sleep duration was associated with low FV consumption	
Buxton et al., 2009 [103]	United States	Motor freight workers	542	Sleep adequacy assessed by “How often during the past 4 weeks did you get enough sleep to feel rested upon waking up?”	6 items of FV (servings/d)	Clustering of workers in trucking terminals through inclusion of terminal as a random effect	Adequate sleep was associated with more servings of FV	Several confounders were not adjusted for in the model
Baron et al., 2011 [104]	United states	Adults recruited from the community	52 adults aged 18–71 years	Sleep timing assessed using logs and wrist actigraphy for 7 d	Food log in which participants recorded all food and drinks consumed for a 7 d period	Age and sleep duration	Sleep timing was independently associated with FV consumption. Later sleep timing was associated with fewer servings of FV	Exclusion criteria did not include shift workers, no participants reported shift work but this could cause report bias. Morning type diurnal preference participants were excluded providing no comparison with evening type participants
Kim et al., 2011 [105]	United States	Women aged 35–74 years	27,983	Subjective report of sleep duration	Eating pattern was self-reported and conventional eating and snack dominance scores were calculated, HEI calculated from FFQ	Age, race, income, education, employment, marital status, children, BMI, menopause status, smoking, alcohol, physical activity, health status, and stress	FV consumption (servings/d) were different among the four quartiles of conventional eating score. Short and long sleepers showed preponderance of snacks over meals related to lower intakes of FV	May have over adjusted and did not adjust for total energy intake
Haghighatdoost et al., 2012 [106]	Iran	Female university students aged 18–28 years	410	Subjective report of sleep duration. Sleep duration were categorized based on the tertiles of sleep duration: <6 h, 6–8 h, and >8 h	168 items of FFQ. Diet diversity and HEI were calculated	No adjusted variables because the study was comparing dietary intake between tertiles of sleep duration	Consumption of fruits was significantly lower in the lowest tertile (<6 h) compared to the highest tertile (>8 h). Diversity scores of FV were significantly lower among participants in the lowest tertile	
Hoefelmann et al., 2012 [122]	Brazil	Workers part of a national survey	47,477	Self-report of FV (servings/week)	Subjective report of sleep quality	Socio-demographic indicators negative perception of health, wellbeing, stress, and self-reported morbidities	Inadequate FV consumption was associated with poor sleep quality	
Mosca and Aggarwal, 2012 [107]	United States	Men older than 40 years and women older than 50 years	371	Subjective report of sleep duration and snoring (yes, no).Sleep duration categorized to (<6 h/d) and (≥6 h/d)	<5 or ≥5 servings/d of FV	Age, sex, ethnicity, and marital status	No difference was shown between sleep duration categories and FV consumption. Snoring was associated with consuming less than 5 servings/day of FV	Assessment method of FV was not mentioned, may be self-report using a standardized questionnaire
Tu et al., 2012 [89]	China	Chinese women aged 40–70 years from the Shanghai Women’s Health Study	68,832	Subjective report of sleep duration. Sleep duration categorized; ≤4 h, 5 h,6 h, 8 h, 9 h, and ≥10 h	FFQ	Age, education level, occupational status, history of night-shift work, annual income, menopausal status, marital status, and number of live births	Fruit intake was inversely associated with short sleep duration. FV consumption was not associated with long sleep	Exposure and outcome not clearly stated
Beydoun et al., 2014 [123]	United States	Adults aged 20–85 from the NHANES	2459	Two 24-h dietary recalls. FV consumption (cup equivalent/d)	Subjective report of sleep	No adjustment	Very short, short and long sleepers consumed less FV compared to normal	
Katagiri et al., 2014 [124]	Japan	Middle-aged female workers aged 34–65 years	3129	151-item self-administered diet history questionnaire	PSQI	Physical activity, CES-D score, employment, smoking, and BMI	High intake of vegetables were associated with good sleep quality	Analyses was not adjusted for several potential confounders e.g., age, total energy intake, SES, and ethnicity
Mota et al., 2014 [98]	Brazil	Resident physicians	72	Sleepiness assessed using the ESS. Sleep quality assessed using PSQI	Food diary for 3 non-consecutive days. FV consumption calculated using AHEI	Age and BMI	FV consumption were not correlated with ESS and PSQI	Exposure/outcome not clearly stated. Pearson correlation was used, does not provide predictions [130]. Analyses were not adjusted for several potential confounders
Chang et al., 2015 [108]	United States	Overweight and obese pregnant women	213	Sleep was assessed by PSQI	7 items of FV assessed by the Rapid Food Screener	Not stated may be due to the use of Pearson correlation and path analyses (to investigate the mediating roles)	Sleep duration and sleep quality were not associated with FV intake in three trimesters. SOL was related to FV in the first and third trimester	
Grandner et al., 2015 [125]	United States	Nationally representative adults	323,047	Daily servings of FV from the BRFSS	Self-report of perceived insufficient sleep	Not clear	Consuming <1 or 1–3 servings of FV was not associated with insufficient sleep	Adjusted variables were not clearly reported
Kurotani et al., 2015 [126]	Japan	Workers aged 18–70 years	2025	52-item diet history questionnaire. Healthy DPs included vegetables, mushrooms, potatoes, seaweeds, soy products, and eggs	Subjective report of seep duration, difficulty initiating and maintaining sleep, and sleep quality	Age, sex, site, shift work, employment, marital status, BMI, smoking, alcohol, physical activity, diabetic treatment, energy intake, skipping meals, habitual snacking at night	An inverse association was found between the healthy DPs and difficulties falling asleep at least once a week and persisted after excluding participants with severe depressive symptoms	May have over adjusted
Mossavar-Rahmani et al., 2015 [99]	United states	Hispanic/Latino participants aged 18–74 years	11,888	Subjective report on sleeping and waking times. Sleep duration categorized: short ≥3 h and <6 h, intermediate >6 h and ≤9 h, long >9 h and ≤ 14 h	Two 24-h dietary recalls. AHEI-2010 scores for diet quality	Age, sex, Hispanic/Latino background, income, employment status, education, depressive symptomology, and years lived in the US	Short sleepers had a lower quality diet compared to intermediate sleepers with significantly lower intakes of vegetables. Long sleepers had lower intakes of FV compared to intermediate sleepers	Exposure and outcome not clearly stated
Patterson et al., 2016 [109]	United Kingdom	Adults aged 40–69 from the UK Biobank	439,933	Subjective report of sleep duration categorized; very short ≤4 h, short 5–6 h, adequate 7–8 h, and long ≥9 h	Self-report of FV consumption for the previous year	Age, sex, ethnicity, attended college, and employment	Longer sleep duration was negatively associated with daily fruit intake, but positively associated with vegetable intake	FV consumption for the previous year may cause over/under reporting
Quick et al., 2016 [127]	United States	College students aged 18–24 years	1252	FV consumption over the past month (cups/day)	PSQI. Sleep duration categorized; <7 h/night, 7–8 h/night and ≥8 h/night	Sex, ethnicity, work time pressures, negative affect, and sleep disturbances	No difference was found in FV consumption between sleep duration groups	
Silva et al., 2016 [110]	Brazil	Students aged 18–39	204	Perceived sleep debt calculated (preferred weekday sleep duration-self reported weekday sleep duration)	FFQ	Age, BMI, and sex	FV consumption were not associated with perceived sleep debt	
Xiao et al., 2016 [111]	United States	Women within 5 years of childbirth aged 20–44 years	896	Subjective report of sleep duration. Sleep duration was categorized to ≤6 h, 7–8 h, and long ≥9 h	Diet was assessed by two 24-h dietary recalls. Diet quality was measured by HEI-2010	Age, ethnicity, education, marital status, poverty income ratio, weight status, years after recent childbirth, smoking, physical activity, depressive symptoms, history of breastfeeding, and diagnoses of chronic diseases	Short sleep duration was not associated with FV consumption. Long sleep duration was associated with lower consumption of total fruit and whole fruit	May have over adjusted
Doo and Kim 2017 [112]	Korea	Pre and post-menopausal women	17,841	Subjective report of sleep duration. Sleep duration categorized to short (≤6.9 h/d) and adequate (≥7 h/d)	One 24-h recall	Age, education, household income, diseases, smoking, alcohol, and physical activity	No differences were observed in FV consumption by sleep duration	
* Duke et al., 2017 [80]	**United States**	**Pregnant**	**2942**	**FV consumption, 4 questions from the BRFSS**	**Subjective report of sleep duration**	**Age, ethnicity, education, exercise, marital status, income, employment**	**Orange and green vegetables were inversely associated with sleep duration. Total FV were not associated with sleep duration. Odds of meeting or exceeding sleep recommendation increased with each unit increase in total FV (OR = 1.05 95% CI 1.003, 1.092)**	**Recall of FV intakes was for the past month which is based on memory and may cause over or underreporting**
Kleiser et al., 2017 [113]	Bavaria, Germany	Bavarian adults aged ≥18	814	PSQI	Three 24-h dietary recalls (2 weekdays, 1 weekend day)	Age, sex BMI, education, smoking physical activity, TV/PC use, and season	Sleep duration was not associated with FV consumption	
Mossavar-Rahmani et al., 2017[114]	United States	Hispanic/Latino participants aged 18–74 years from 4 US cities	2140	Sleep measured by actigraphy for 7 consecutive days. Sleep duration categorized; short (<6 h), intermediate (= 6 and <8 h) and long (≥ 8 h). Sleep fragmentation index calculated	Two 24-h dietary recalls. AHEI-2010 scores for diet quality	Age, sex, site, ethnic background, employment depression, and log daily energy intake	Whole fruit intake differed between sleep duration groups with lowest intakes in short sleepers. Sleep efficiency was positively associated with whole fruit intake and sleep fragmentation index was negatively associated with whole fruit intake	
Pérez-Rodrigo et al., 2017 [128]	Spain	Adults aged 18–64	1617	24-h diet recall, a 3-day food record aided by a tablet device. Four DPs identified; traditional (high in FV), Mediterranean (high in FV), snack and dairy	Subjective report of sleep duration. Three lifestyle patterns identified; “Mixed diet-physically active-low sedentary lifestyle pattern”, a “Not poor diet-low physical activity-low sedentary lifestyle pattern”, and a “Poor diet-low physical activity-sedentary lifestyle pattern”	Age	Sleep duration differed between the 3 lifestyle patterns in men and women. In both men and women, mean sleep duration was the highest in the “Not poor diet-low physical activity-low sedentary lifestyle pattern”	Two DPs were identified with high intakes of FV. Analyses was not adjusted for several potential confounders
Potter et al., 2017 [90]	United Kingdom	Adults aged 19–65 years from the NDNS	1615	Subjective report of sleep duration	4-day food diary	Age, sex, smoking, ethnicity, and SES	Sleep duration was not associated with FV consumption	Did not adjust for total energy intake. Non-linear associations not explored between sleep and diet
Timmermans et al., 2017 [115]	Europe	Adults	5900	Subjective report of sleep duration	FFQ	Age, sex, education and self-rated health	Longer sleep duration was associated with lower fruit consumption	
Van Lee et al., 2017 [100]	Singapore	Pregnant women	497	PSQI	One 24-h recall at 26–28 weeks of gestation. HEI-SGP to measure diet quality. DPs included FV and white rice pattern	Alcohol, physical activity, household income, education, ethnicity, energy intake, age, and gravidity	Good sleep quality was associated with better diet quality and greater adherence to the FV and white rice pattern compared to poor sleep quality	Exposure and outcome not clearly stated
Wang et al., 2017 [129]	China	Older adults aged 60–79 years	4115	Inadequate fruit intake was defined as adults who ate fruit less than three times per week	Subjective report of sleep duration. Sleep duration was categorized to <7 h/d, 7–8 h/d and >8 h/d	All independent variables of socio-demographic and lifestyle variables were included in the same model thus adjusting for each other	Inadequate intake of fruits was positively associated with short and long sleep durations	The definition of inadequate fruit was not based on a reference
Gebski et al., 2018 [116]	Polish adults	Adults aged 21–65 years	1007 adults	Subjective report of sleep duration	Frequency of consumption of selected food groups including FV. Five DPs were derived including FV pattern and FV juices	Age, education and place of residence	In weekdays, short sleep duration was associated with lower odds of FV DP in men. In weekends, short sleep duration was associated with higher odds of FV DP in women	Analyses was not adjusted for several potential confounders
* Lee et al., 2018 [81]	**China**	**Older adults aged ≥65 years**	**5911**	**Subjective report of the frequency of FV consumption**	**Subjective report of sleep duration and quality. Sleep duration categorized; short (<7 h), recommended (7–8 h) and long (>8 h)**	**Age, sex, marital status, education, alcohol, smoking, exercise, household income, community, and province**	**Frequent FV consumption were associated with better sleep quality. Less frequent FV consumption was associated with short sleep and long sleep compared to the reference **	**Did not test for non-linear associations. Dietary recall may cause over or under reporting**
* Noorwali et al., 2018 [84]	**United Kingdom**	**Adults aged 19–65 years from the National Diet and Nutrition Survey**	**1612**	**Subjective report of sleep duration categorized to short (<7 h/d), reference (7–8 h/d) long (>8 h/d)**	**4-day food diaries. Foods containing FV were disaggregated into their components to help assess total FV. **	**Age, sex, SES, smoking, ethnicity, and total energy intake**	**Sleep duration was non-linearly associated with FV consumption with short and long sleepers having lower intakes compared to the reference group**	**Assessed non-linear associations and used FV biomarkers**
Patterson et al., 2018 [117]	United Kingdom	Adults aged 40–69 enrolled in the UK Biobank	438,933	Subjective report of sleep duration. Sleep duration was categorized to ≤6 h/d, 7–8 h/d and ≥9 h/d	FFQ. Variables combined and a binary variable created to (<5 servings/d, ≥5 servings/d)	Age, sex, ethnicity, employment, shift work, education, urban vs. rural residence	Long sleepers with had a 62% higher odds of eating <5 servings/d of FV compared with adequate sleepers	Sleep duration and chronotype were used together as independent variables suggesting interactive effects
Peltzer et al., 2018 [118]	South Africa	Participants aged ≥ 40 years	4725	Subjective report of sleep duration. Sleep duration categorized to <7 h/d, 7–8 h/d and ≥9 h/d	Self-report of FV consumption. Inadequate FV consumption: having <5 servings/day	Not stated	Consumption of <5 servings/day of FV were associated with higher odds of short sleep duration	Authors state adjusted multinomial logistic regression but did not state the confounders
Tan et al., 2018 [119]	Germany and Netherlands	Participants aged 20–85 years	790	Subjective report of restful sleep and sleep quality	Self-report of FV consumption. “During the last weeks, did you eat five portions of FV per day?” The answers were based on a five-point Likert scale	Age, sex, BMI, country of origin, employment status, marital status, and education	Restful sleep was not associated with FV consumption however, in combination, restful sleep, physical activity, and FV intake were associated with increased sleep quality	
Vézina-Im et al., 2018 [120]	Canada	Women of child bearing age 18–44 years	9749	Subjective report of sleep duration and quality. Sleep duration was categorized to <7 h/night and ≥ 7 h/night	6-item questionnaire to assess FV consumption	No adjustment	FV intake was associated with higher odds of having adequate sleep duration and quality sleep	
Vézina-Im et al., 2018 [121]	Canada	Women of child bearing age 18–44 years	9749	Subjective report of sleep duration and quality. Sleep duration was categorized to <7 h/night and ≥ 7 h/night	6-item questionnaire to assess FV consumption	Age, ethnicity, education, household income, marital status, employment, parity, region, season, mood disorder, FV intake, physical activity, smoking, and alcohol	FV consumption was included as an adjustment between sleep duration and quality with BMI. FV consumption was not associated in the relationship between sleep duration and quality with BMI ≥25	This study assessed the association between sleep duration and quality with BMI adjusting for several covariates including FV intakes
**Prospective studies**								
Imaki et al., 2002 [78]	Japan (6 year follow-up)	Male employees aged 20–59 years	2000	Multiple choice questionnaire: hours of sleep, (1) ≤6 h, (2) 6.1–8.9 h, (3) ≥9 h	7 items of dietary habits including vegetable intakes in the diet (1) ample (2) none	No adjustment	The percentage of participants who slept 6 h or less consumed less vegetables compared to 6.1–8.9 h during the 6-year period of study	This study did not use any analyses for prediction such as regression analyses and only compared the intakes using percentages
Huang et al., 2013 [79]	Taiwan (10 year follow-up)	Elderly aged ≥65 years	1865	Subjective report of sleep quality categorized; poor, fair or good	24-h dietary recall and FFQ. Dietary diversity score derived from 6 items including FV	Age, education, BMI, physical activity, and use of sleeping pills	Female poor sleepers consumed fewer vegetables compared to fair or good sleepers. Dietary diversity score and sleep quality interacted and modulated mortality with sex differences	
* Noorwali et al., 2018 [82]	**United Kingdom**	**Middle aged women from the UK Women’s Cohort Study**	**Cross-sectional = 12,159 Prospective = 463**	**Subjective report of sleep duration categorized to short (≤6 h/d) recommended (7–9 h/d) long (≥9 h/d)**	**4-day food diaries**	**Age, SES, smoking, ethnicity, and total energy intake**	**Sleep duration was non-linearly associated with FV consumption in cross-sectional and prospective analyses with those sleeping the recommended 7–9 h having the highest intakes**	**First prospective study. Assessed non-linear associations and used FV biomarkers**
* Noorwali et al., 2018 [83]	**United Kingdom **	**Middle aged women from the UK Women’s Cohort Study**	**13,958**	**FV items from FFQ and their polyphenol content matched from Phenol Explorer database**	**Subjective report of sleep duration**	**Age, SES, smoking, ethnicity and total energy intake**	**FV consumption and their polyphenol content were inversely associated with sleep duration **	**First prospective study to examine the association between polyphenols from FV and sleep duration **
**Sleep restriction and extension studies**								
Spiegel et al., 2004 [67]	United States	Healthy young men	12	Men were assigned to either 4 h of sleep for 2 consecutive nights or 10 h of sleep for 2 consecutive nights	Participants were provided with standard hospital meals and completed a visual analogue scale for hunger and appetite for various food categories including FV	No adjustment	Appetite rating for FV increased following sleep restriction by 17% (*p* = 0.07) for fruit and fruit juices and 21% for vegetables (*p *= 0.02) compared to sleep extension	Short intervention period and small sample size
**Sleep restriction and extension studies**								
Heath et al., 2012 [68]	Australia	Healthy males	24	Participants lived 12 consecutive days in a sleep laboratory. 14 participants were sleep restricted to 4 h (severe), 10 participants were restricted to 6 h of sleep (moderate)	Participants were served 3 meals and 5–6 snacks daily. Snacks included 3 categories; sweet, savoury and healthy (1 piece of fresh fruit and 1 packet of 40 g of dried fruit and nuts)	No adjustment	No effects of sleep restriction were found on healthy snack consumption	Short intervention period and small sample size
Spaeth et al., 2014 [69]	United States	Healthy adults aged 21–50 years	44	In laboratory sleep restriction to 4 h (04:00–08:00 a.m.) for 5 consecutive nights. Participants wore actigraph	Participants selected their meals and snacks by choosing from various menu options, selecting additional food and drink available in the laboratory suite	Age	Calories consumed from FV and salad did not differ between baselines and sleep restriction	
**Sleep extension studies**								
Tasali et al., 2014 [70]	United States	Overweight young adults reporting sleep <6.5 h/d	10	Habitual sleep was followed for 1 week and intervention was extending sleep to 8.5 h for 2 weeks by behavioral counselling on sleep hygiene	Desire for various foods including FV was assessed using visual analog scales	No adjusted variables	Extended sleep did not change the desire for FV	No control group. Short intervention period and small sample size
**Fruit intervention studies**								
* Garrido et al., 2009 [71]	**Spain**	**Young, middle-aged, and elderly**	**18**	**Powdered freeze-dried nutraceutical product diluted in 125 mL water equivalent to 141 g Jerte Valley cherries, consumed twice a day for 3 consecutive days**	**Sleep was assessed by actigraphy. Participants wore it 3 days before the trial, during 3 days of trial, and 1 day afterwards.**	**No adjusted variables**	**After intervention, sleep duration increased compared to baseline. Immobility increased and nocturnal activity decreased in young and elderly compared to baseline**	**No control group. Short intervention period and small sample size**
* Garrido et al., 2010 [72]	**Spain**	**Middle-aged and elderly Caucasian**	**12**	**200 g of 7 different cultivars of cherries twice a day for three days**	**Wrist actigraphy wore 3 days before the trial and during 3 days of the trial**	**No adjusted variables**	**Sleep duration and immobility increased after intervention, the number of awakenings, sleep latency, and nocturnal activity decreased**	**No control group. Short intervention period and small sample size**
* Pigeon et al., 2010 [73]	**United States**	**Healthy older adults aged ≥65 years with insomnia**	**15**	**Tart cherry juice blend or placebo consumed for 2 weeks twice a day in the morning between 8:00–10:00 a.m. and in the evening 1–2 h before bedtime**	**Sleep was assessed by an ISI and sleep diaries**	**No adjusted variables**	**Within groups, tart cherry juice improved ISI, SOL, sleep duration, sleep efficiency and wake after sleep onset. Between groups, tart cherry juice reduced the ISI score and wake after sleep onset with no difference in SOL, sleep duration, and sleep efficiency**	**Short intervention period and small sample size**
* Lin et al., 2011 [74]	**Taiwan**	**Participants self-reporting sleep disturbance aged 20–55 years**	**24**	**Two kiwifruits consumed 1 h before bedtime for 4 weeks**	**CPSQI, sleep diary, and actigraph**	**No adjusted variables**	**After intervention, Actigraph and sleep diary showed that sleep duration and efficiency increased compared to baseline. Sleep diary showed a decrease in CPSQI score, waking time after sleep onset, and SOL**	**No control group. Participants included only 2 males and 22 females. Kiwifruit consumption on sleep may differ by sex**
* Howatson et al., 2012 [75]	**United Kingdom**	**Healthy adults**	**20**	**Participants consumed a tart cherry juice concentrate or placebo for 7 d**	**Sleep quality recorded by actigraphy and online subjective sleep diaries were collected**	**No adjusted variables**	**Sleep diary showed that cherry juice intake decreased napping time. Actigraphy showed that cherry juice increased time in bed, sleep duration, and sleep efficiency**	**Short intervention period and small sample size**
* Garrido et al., 2013 [76]	**Spain**	**Young middle-aged and elderly**	**30**	**Jerte Valley cherry based product (JVCP) consumed twice a day as lunch and dinner desserts for 5 d or a placebo **	**Sleep was assessed by actigraphy. Participants wore it 5 d before the trial, during 5 d of trial and 5 d afterwards.**	**No adjusted variables**	**JVCP increased sleep duration and immobility in young, middle-aged and elderly compared to baseline and placebo. JVCP increased sleep efficiency in elderly compared to baseline. SOL decreased in middle-aged and elderly**	**Short intervention period and small sample size**

Legend: AHEI (Adapted Healthy Eating Index); AHEI-2010 (Alternative Healthy Eating Index); ANOVA (analyses of variance); BMI (body mass index); BRFSS (Behavioral Risk Factor surveillance System); CES-D (Centre for Epidemiological Studies Depression scale); CPSQI (Chinese version of the Pittsburgh Sleep Quality Index); CVD (cardio vascular disease); d (day); DPs (dietary patterns); ESS (Epworth Sleepiness Scale); FFQ (food frequency questionnaire); FV (fruit and vegetable);g (gram); h (hour); HEI (Healthy Eating Index); HEI-SGP (Healthy Eating Index for Pregnant women in Singapore); ISI (Insomnia Severity Index); JVCP (Jerte Valley cherry based product); *n* (number); NDNS (National Diet and Nutrition Survey); NHANES (National Health and Nutrition Examination Surveys); OR (odds ratio); PSQI (Pittsburgh Sleep Quality Inventory); Ref (reference); SES (socio-economic status); SOL (sleep onset latency). * BOLD row, Key paper with main objective assessing the association between sleep measures and fruit and vegetable consumption.

**Table 3:** Summary of animal and in vitro studies [references] assessing the effects of polyphenols on sleep and their potential mechanisms.

	Potential Mechanism Assessed				Sleep Assessed	
	Serotonin 1A Receptor	GABA Receptors	Circadian Rhythms	Clock Gene Expression	Sleep/Wake Cycles	Sleep Duration
In vivo	[202]	[203,204,205,206,207]	[208,209,210]	[211,212,213,214,215]	[202,204,205,206,207,216,217,218,219]	[202,203,205,206,207,216,217,218,220]
In vitro			[221]	[222]		
**Polyphenol**						
Flavonoids	[202]	[205]	[221]		[202,205]	[202,205,220]
Resveratrol			[209,210]	[212,213]	[219]	
Phenolic acids						[220]
GSPEs				[211,214,215]		
Phlorotannins *		[203,204]			[204]	[203]
Triphlorethol A*					[216]	[216]
Red cabbage extracts					[217]	[217]
Kiwifruit extracts		[206]			[206]	[206]
Romaine lettuce					[218]	[218]
Tea polyphenols		[207]		[222]	[207]	[207]
Cherry			[208]			

Legend: GSPEs (Grape seed proanthocyanidins extracts), GABA (γ-aminobutyric acid), * Sea weed polyphenols.

**Table 4:** Adult human interventional studies exploring the effects of polyphenols on sleep.

Author, Year (REF)	Study Type	Population	Sample *n*	Polyphenol Intervention	Intervention Period	Findings Reported on Polyphenol Effect on Sleep
Kuratsune et al., 2010 [233]	Double-blind, placebo-controlled, cross-over	Healthy men with mild sleep complaint	21	Crocetin, active carotenoid	Two intervention periods of 2 weeks each separated by a 2-week washout	Actigraphic data showed a reduction in the number of wakening episodes compared to placebo. Subjective data showed improvement in sleep quality
Wightman et al., 2015 [234]	Randomized, double-blind, placebo-controlled, parallel	Adults aged 18–30 years	60	Resveratrol	28 days	No effect on PSQI score or its seven factors
Park et al., 2017 [235]	Double-blind, placebo-controlled, cross-over	Healthy adults	9	Chlorogenic acids, most abundant polyphenol in coffee	5 days	Shortened SOL compared with the control with no effect on sleep architecture
Herrlinger et al., 2018 [231]	Double-blind, placebo-controlled, parallel	Older adults with age associated memory impairment	90	Spearmint extract containing 24% total polyphenols	90 days	Improved the ability to fall asleep, alertness, and behavior following wakefulness compared to controls
Um et al., 2018 [236]	Randomized, double-blind, placebo-controlled, parallel	Adults with subjective sleep disturbances	24	Phlorotannin	One week	Sleep duration increased compared to placebo, however no effects were shown on the total PSQI score
Romain et al., 2017 [237]	Randomized, double-blind, placebo-controlled, parallel	Overweight and obese adults	33	Holisfiit^®^, a polyphenol-rich extract-based food supplement developed from FV	16 weeks	Awakening during the night improved by 38%, total sleep duration by 50%, and sleep quality by 43% compared to baseline and subjective sleep complaints improved significantly compared to controls
Uddin et al., 2018 [238]	Randomized, double-blind, placebo-controlled, cross-over	Pre-hypertensive adults	12	Fruitflow^®^ supplements, tomato extract	24-h period	Both systolic and diastolic blood pressure were lower after FruitFlow^®^ consumption compared to placebo in the wake period whereas during the sleep period, the effect was only shown for systolic blood pressure only
Grassi et al., 2016 [239]	Randomized, double-blind, cross-over	Healthy adults	32	Flavanol-rich chocolate	Consumption of (high or poor flavanol chocolate bars) after one night of total sleep deprivation	High-flavanol chocolate bar reduced high systolic and diastolic blood pressure caused by sleep deprivation compared to low-flavanol chocolate bar consumption
Bigelman et al., 2011 [240]	Randomized, double-blind, placebo-controlled, cross-over	Healthy adults conducting military physical training	58	Quercetin	6 weeks	No effects on sleep quality

Legend: PSQI (Pittsburgh Sleep Quality Inventory), SOL (sleep onset latency), REF (reference), *n* (number).

**Table 5:** Recommendations for future studies investigating the relationship between sleep and FV consumption.

Recommendations
1. Consider individual differences of sleep by exploring optimal sleep duration in a laboratory.
2. Explore the effects of sleep disruption on FV consumption based on genetic disparities.
3. Explore the effects of different FV items (individually and combined) on sleep measures.
4. Compare the effects of FV consumption at specific time points on sleep measures.
5. Explore the effects of other FV components (e.g., micronutrients, moisture, fiber, polyphenols, antioxidants and offsetting energy intake) on sleep measures and architecture.
6. Identify a biomarker of polyphenol consumption to be used in studies exploring the relations between sleep and FV.
7. Consider food matrix by comparing the effects of polyphenol administration between participants fasting, consuming a complex meal and consuming FV items.
8. Controlling for other sources of polyphenols such as coffee, tea, red wine, soy, and chocolate.
9. Group FV items based on similarity of total antioxidant and explore their effects on sleep and compare their effects with antioxidants from supplements.
10. Identify objective markers of antioxidant intake and compare their levels across different sleep durations, quality, and chronotypes.
11. In RCT exploring the effects of FV on sleep, selection of FV items that undertaken similar food processing and handling methods to account for antioxidant preservation is necessary.
12. Investigate the effects of sleep timing, taking chronotype into account, on FV consumption.
13. Non-linear relationships between FV and sleep need to be considered in observational and sleep restriction/extension studies.
14. Explore the relations between sleep and FV consumption in other populations such as; different ethnicities, elderly, clinical populations, shift workers, and less-developed countries.
15. Study whether FV consumption is substituted with convenient desserts and sweets in response to sleep disruption.
16. Identify how to optimize exercise protocols to increase FV consumption and improve sleep.
17. Compare the effects of sleep hygiene education and digital cognitive behavioral therapy on FV consumption in sleep extension studies.
18. Identify the role of stress and self-control in the relationship between sleep and FV consumption.

Legend: FV (fruit and vegetable), RCT (randomized controlled trials).

## Supplementary Materials

The following are available online at https://www.mdpi.com/2072-6643/11/6/1382/s1, Table S1: Search terms used for literature review.
