# Supplementary material for: Bridging the Reciprocal Gap between Sleep and Fruit and Vegetable Consumption: A Review of the Evidence, Potential Mechanisms, Implications, and Directions for Future Work
Source: Nutrients. 2019 Jun 19;11(6):1382. doi: 10.3390/nu11061382 (PMC6627504; doi:10.3390/nu11061382)
Supplement: Supplementary file 1 [file nutrients-11-01382-s001.pdf]

Table S1. Search terms used for literature review

1. SLEEP/
2. sleep.tw.
3. or/1-2 [sleep]
4. exp Fruit/
5. exp Citrus/
6. exp VEGETABLES/
7. or/4-6 [fruit or vegetables]
8. (food adj2 intake*).tw.
9. (dietary pattern or dietary habit*).tw.
10. food consumption.tw.
11. Food Habits/
12. Food Preferences/
13. Nutritional status/
14. Nutritional assessment/
15. Diet/
16. Health behavior/
17. diet*.tw.
18. Eating/
19. eating.tw.
20. or/8-19 [diet terms]
21. 7 or 20 [fruit or veg or diet]
22. 3 and 21 [sleep and fruit or veg or diet]
23. (exp adolescent/ or exp child/ or exp newborn/) not ((exp adult/ or exp aged/ or exp middle aged/) and (exp adolescent/ or exp child/ or exp newborn/))
24. 22 not 23 [adult only results]
25. exp animals/ not exp humans/
26. 24 not 25 [human only studies]
27. exp BIOMARKERS/
28. biomarkers.tw.
29. or/27-28 [biomarkers]
30. 3 and 29
31. exp POLYPHENOLS/
32. polyphenols.tw.
33. or/31-32 [polyphenols]
34. 3 and 33
